# The complete mitochondrial genome of *Nothochrysa sinica* (Neuroptera: Chrysopidae: Nothochrysinae) with a phylogenetic analysis of Chrysopoidea

**DOI:** 10.1080/23802359.2021.1926361

**Published:** 2021-05-13

**Authors:** Ruyue Zhang, Yunlong Ma, Fan Fan, Shuo Geng, Yuyu Wang, Xingyue Liu

**Affiliations:** a College of Plant Protection, Hebei Agricultural University, Baoding, China; b Department of Entomology, China Agricultural University, Beijing, China

**Keywords:** *Nothochrysa sinica*, mitochondrial genome, phylogeny

## Abstract

The complete mitochondrial (mt) genome of *Nothochrysa sinica* Yang (Neuroptera: Chrysopidae: Nothochrysinae) is reported in this work. It represents the first complete mt genome of the subfamily Nothochrysinae. The whole mt genome is 16,166 bp long and contains 13 protein-coding genes (PCGs), 22 transfer RNA genes (tRNAs), 2 ribosomal RNA genes (rRNAs), and an AT-rich region. Most PCGs used the typical ATN as initiation codons. The AT-rich region is 1,271 bp long with 90.24% of A + T. The results show that *N. sinica* is closely related to *N. californica*. Chrysopidae was demonstrated monophyletic being the sister group to Hemerobiidae. Within Chrysopidae, the sister-group relationship between Nothochrysinae and Apochrysinae was supported and together being the sister group to Chrysopinae.

*Nothochrysa sinica* Yang, 1986 belongs to Nothochrysinae (Neuroptera: Chrysopidae), which comprises only 28 species in 9 genera distributed in temperate regions (Yang 1986; Tauber and Faulkner 2015). Nothochrysinae is more common in fossil records compared with other subfamilies (Nel et al. 2005). With the development of molecular biology technology, mitochondrial (mt) genome has been widely used in the study of insect evolution and phylogeny due to its rapid evolutionary speed, maternal inheritance and rare recombination (Cameron 2014). However, there is only one partial mt genome (*N. californica*) from Nothochrysinae published on GenBank.

Herein, we sequenced and annotated the complete mt genome of *N. sinica* (GenBank accession number: MW699775). The specimen used for DNA extraction was collected by Yingnan He at Jialingjiangyuan, Feng Country, Baoji City, Shaanxi Province, China (106°56′52″E, 34°15′5″N). The voucher specimen was deposited in Hebei Agricultural University Museum (hebau_entmus@126.com, No. CHR001). Total genomic DNA was extracted from thoracic muscle of a single adult using the DNeasy Blood & Tissue kit (QIAGEN, Germany). Genomic DNA was sequenced using the Illumina NovaSeq 6000 platform with 150 bp paired-end reads. Raw reads were checked by FastQC 0.11.9 (Andrews 2020) and low-quality reads were filtered by Trimmomatic 0.32 (Bolger et al. 2014). The mt genome was assembled by IDBA-UD 1.1.3. (Peng et al. 2012) and further annotated using the MITOS Web Server (Bernt et al. 2013) as well as manually proofed.

This mt genome of *N. sinica* is a double-stranded circular molecule with 16,166 bp long containing 22 transfer RNA genes (tRNAs), 13 protein-coding genes (PCGs), 2 ribosomal RNA genes (rRNAs) and an AT-rich region, which are typical in metazoan mt genomes (Wolstenholme 1992). Among them, 23 genes are located on the main chain (J) and 14 genes are located on the minority chain (N). The base composition of the whole mt genome is 38.64% of A, 41.26% of T, 11.48% of C and 8.62% of G. Almost all of the 13 PCGs of *N. sinica* used the typical initiation codons ATN, except *COI* used TCG as the start codon. There are 4 genes ended with the incomplete stop codon (T-tRNA for *COII*, *ND4*, *ND5* and TA-tRNA for *CytB*) and 9 PCGs terminated with the stop codon TAA. The 22 tRNAs genes vary from 64 bp to 71 bp in length. The length of *rrnL* and *rrnS* is 1,319 bp and 784 bp, respectively. The AT-rich region is located between *rrnS* and *tRNA^Ile^*. It is 1,271 bp in length with an A + T content of 90.24%.

Phylogenetic trees were reconstructed with MrBayes 3.2.2 (Ronquist et al. 2012) and RAxML 8.2.4 (Stamatakis 2014) getting the same topology (Figure 1) based on 13 PCGs. Two species from Mantispidae were used as outgroups. Results showed that *N. sinica* was closely related to *N. californica* with high support values (PP = 1, BS = 100). Chrysopidae was demonstrated monophyletic being the sister group to Hemerobiidae. Nothochrysinae was the sister group to Apochrysinae and together being the sister group to Chrysopinae, which was identical with the previous work (Jiang et al. 2017). The support values of some nodes within Chrysopinae were low indicating that the relationships within Chrysopinae were still confused. More mt genomes are needed to better understand the phylogenetic relationships within Chrysopinae as well as Chrysopoidea in the future.

**Figure 1.**
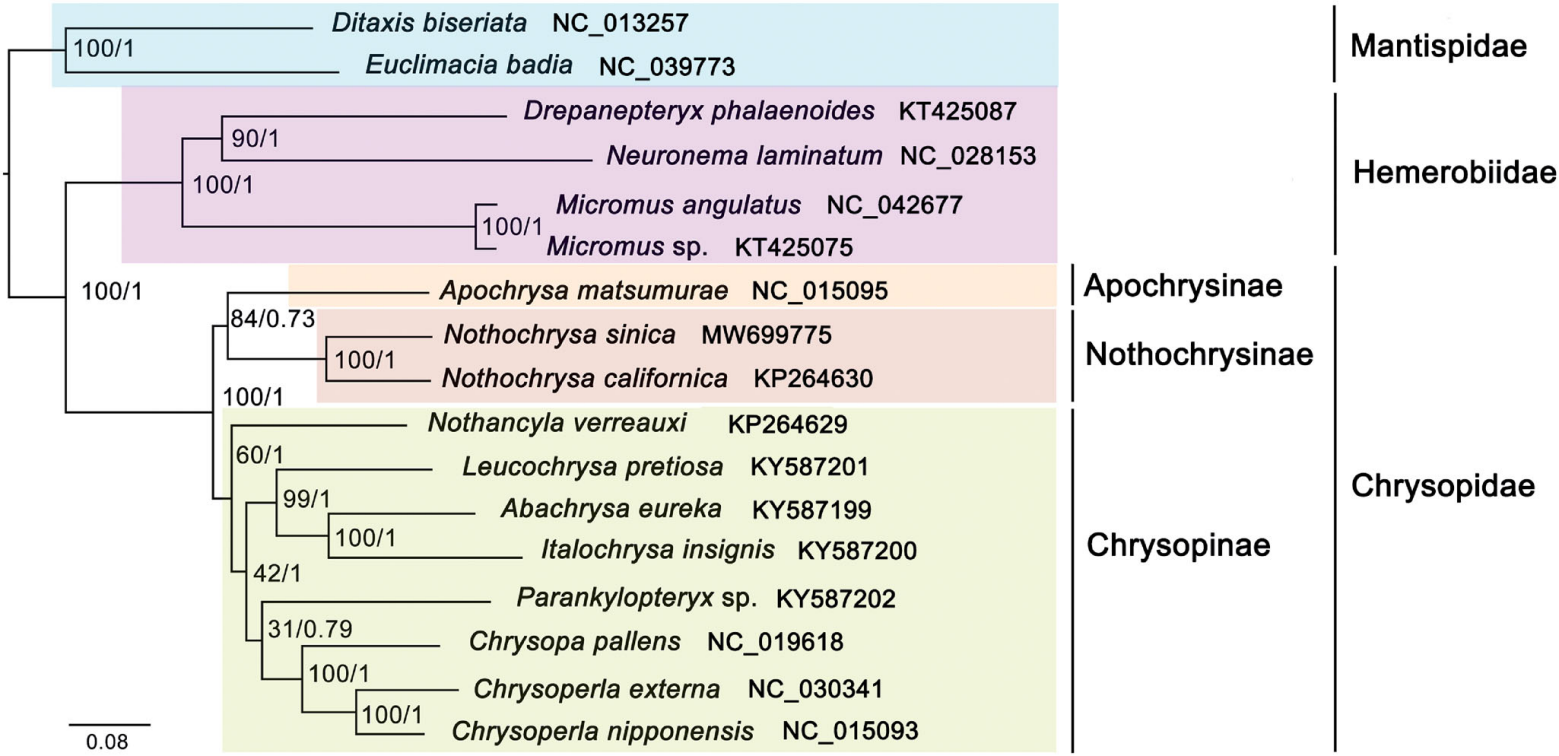
Phylogenetic relationships based on 13 mitochondrial protein-coding genes sequences inferred from RAxML and Mrbayes. The nodal numbers indicate the bootstrap support values (left) and the posterior probability (right). Genbank accession numbers for the sequences are indicated next to the species names.

## Data Availability

The genome sequence data that support the findings of this study are openly available in GenBank of NCBI at https://www.ncbi.nlm.nih.gov under the accession No.MW699775. The associated BioProject, SRA, and Bio-Sample numbers are PRJNA707120, SRR13870182, and SAMN18175023, respectively.
